# Neuromuscular Active Zone Structure and Function in Healthy and Lambert-Eaton Myasthenic Syndrome States

**DOI:** 10.3390/biom12060740

**Published:** 2022-05-24

**Authors:** Scott P. Ginebaugh, Yomna Badawi, Tyler B. Tarr, Stephen D. Meriney

**Affiliations:** Department of Neuroscience, University of Pittsburgh, Pittsburgh, PA 15260, USA; spginebaugh@gmail.com (S.P.G.); y.badawi@pitt.edu (Y.B.); tbt9@pitt.edu (T.B.T.)

**Keywords:** active zone, neuromuscular junction, Lambert-Eaton myasthenic syndrome, computational modeling

## Abstract

The mouse neuromuscular junction (NMJ) has long been used as a model synapse for the study of neurotransmission in both healthy and disease states of the NMJ. Neurotransmission from these neuromuscular nerve terminals occurs at highly organized structures called active zones (AZs). Within AZs, the relationships between the voltage-gated calcium channels and docked synaptic vesicles govern the probability of acetylcholine release during single action potentials, and the short-term plasticity characteristics during short, high frequency trains of action potentials. Understanding these relationships is important not only for healthy synapses, but also to better understand the pathophysiology of neuromuscular diseases. In particular, we are interested in Lambert-Eaton myasthenic syndrome (LEMS), an autoimmune disorder in which neurotransmitter release from the NMJ decreases, leading to severe muscle weakness. In LEMS, the reduced neurotransmission is traditionally thought to be caused by the antibody-mediated removal of presynaptic voltage-gated calcium channels. However, recent experimental data and AZ computer simulations have predicted that a disruption in the normally highly organized active zone structure, and perhaps autoantibodies to other presynaptic proteins, contribute significantly to pathological effects in the active zone and the characteristics of chemical transmitters.

## 1. Introduction

Neuromuscular junctions (NMJs) are essential for movement, and thus are essential for normal function. Because of the importance of NMJs to normal functioning, NMJs have evolved a variety of properties that allow them to function under a wide range of conditions. Two such properties are the strength and reliability of NMJs. Here, strength refers to the fact that a typical presynaptic action potential (AP) causes a release of transmitters in excess of what is necessary to lead to the contraction of the postsynaptic muscle fibers. Reliability refers to the fact that the NMJ can repeatedly stimulate, or cause the sustained contraction of, the postsynaptic muscle fibers [1,2].

The release of neurotransmitters from the presynaptic terminals of neurons occurs at specialized regions of the presynaptic membrane called active zones (AZs), which contain docked synaptic vesicles, voltage-gated calcium channels (VGCC), and a variety of structural, membrane-fusion facilitating, and calcium-sensing proteins [3]. The overall function of the NMJ is governed by the probability of chemical transmitter release from hundreds of AZs within each motor nerve terminal. The probability of transmitter release is determined by the presynaptic AP waveform, the gating of presynaptic VGCCs, the sensitivity of calcium sensors on docked synaptic vesicles, and the spatial relationship between VGCCs and docked synaptic vesicles [4,5,6].

Lambert-Eaton Myasthenic Syndrome (LEMS) is a rare autoimmune neuromuscular disease in which the immune system attacks proteins in the AZ structure of the NMJ [7,8]. This attack is thought to reduce the number and organization of VGCCs and other proteins associated with the AZ [9,10]. As a result of this attack, LEMS results in a reduction in the magnitude of neurotransmitter release from the NMJ, which leads to severe muscle weakness for LEMS patients [11].

## 2. Action Potential Triggered Calcium Entry

The AP is often considered as a binary signal that propagates down the motor axon to the nerve terminal, causing a release of neurotransmitters into the synapse upon reaching the nerve terminal [12,13,14]. However, it is clear even from early work on the squid giant axon AP [15,16,17,18,19,20,21] that the size, shape, and conduction velocity of the AP play an important role in regulating communication. Neurons regulate the propagation and shape of the AP with a heterogeneous distribution of ion channels, and the shape of the AP waveform can vary greatly between different neuron types [22] and within different regions of the same neuron [12,23,24,25].

**Figure 1:**
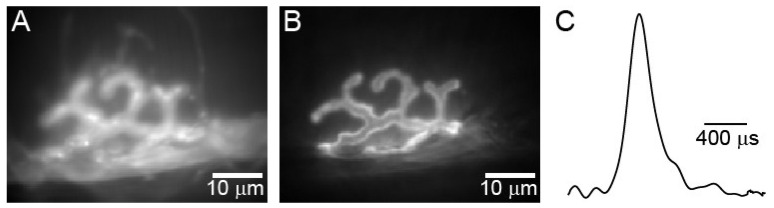
The presynaptic AP waveform at the mouse NMJ is very brief. (**A**) BeRST 1 dye-stained image of a mammalian presynaptic motor nerve terminal. (**B**) Alexa Fluor 488 α-BTX stained image of the same terminal as in A. (**C**) normalized spline of the average presynaptic AP waveform recorded from 11 mouse motor nerve terminals. Adapted from Ojala et al. [26].

Despite the importance of the AP waveform to the function of nerves and synapses, the shape of the AP waveform and how changes in this waveform impact the function of synapses are relatively understudied. This is primarily due to the fact that presynaptic nerve terminals at most synapses are too small to probe with an electrode (with a few notable exceptions). Regarding the mammalian motor nerve terminal, recently, Ojala et al. [26] used voltage imaging to characterize the presynaptic AP waveform. In these studies, they reported that motor-nerve-terminal APs are exceptionally brief, with a full width of 250–350 μs at half maximal amplitude (Figure 1). The brief duration of these AP waveforms is thought to be due in part to the selective expression of voltage-gated potassium channels of the Kv3.3 and 3.4 type [27], and calcium-activated potassium channels [28] within AZs which can increase the rate of repolarization.

These very brief APs are thought to only activate a small fraction of the available calcium channels within AZs. Research using a single pixel optical fluctuation analysis on calcium imaging at the frog NMJ suggests a one-to-one relationship between docked synaptic vesicles and voltage-gated calcium channels in the AZ. Furthermore, calcium imaging found that these AZ voltage-gated calcium channels have only a 0.2 probability of opening during an AP [29,30]. Thus, only a small fraction of voltage-gated calcium channels would be expected to open in each AZ during an AP. This paucity in the total number of voltage-gated calcium channels in each AZ, combined with the low probability that these channels would open during the very brief presynaptic AP provides a mechanism which can explain the low probability of release for synaptic vesicles. The resulting unreliable synaptic vesicle release per AZ is hypothesized to contribute to the reliability of the synapse as a whole by ensuring that, even during repeated stimulation, the synapse will only use a small fraction of the available docked synaptic vesicles and not become depleted of these synaptic vesicles during prolonged and repeated activity [31]. Although this hypothesis is generally accepted, it should be noted that it has not yet been tested experimentally.

## 3. Active Zone Structure and Organization at Healthy Synapses

**Figure 2:**
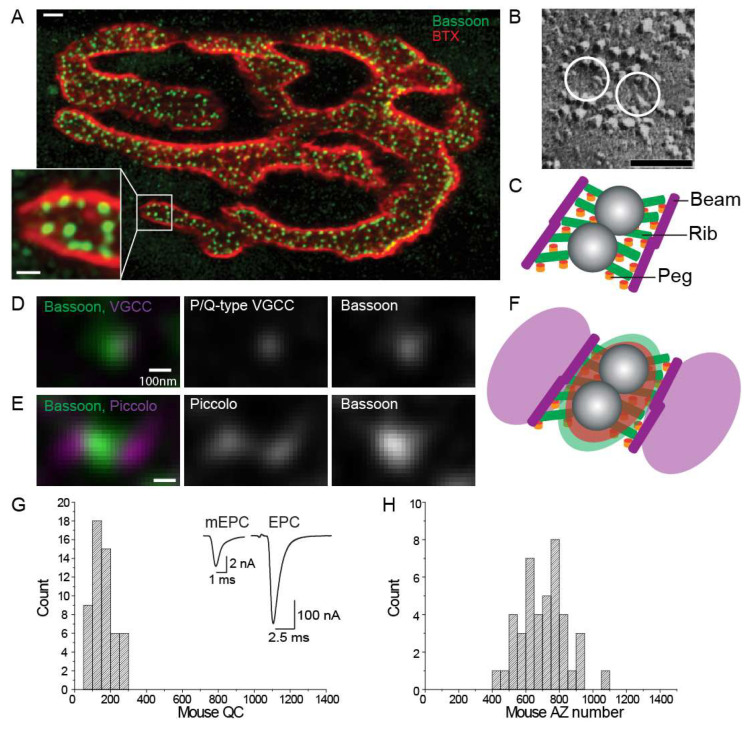
The structure and protein distribution of mouse NMJ AZs. (**A**) A mouse NMJ stained with Alexa-594 α-bungarotoxin (BTX; red) to demonstrate the shape of the NMJ and an Alexa-488 conjugated antibody to identify the bassoon protein the AZs (BSN; green). Inset shows an enlargement of one part of the NMJ to make it easier to visualize the distribution of AZs (green spots). Image adapted from [5]. (**B**) A freeze-fracture replica of an AZ from a mouse NMJ. The hypothesized locations of synaptic vesicles are superimposed as white circles. Scale bar = 50 nm. Adapted from [10,36]. (**C**) Diagram of a single AZ from a mouse NMJ based on electron microscope tomography data [36]. Diagram shows docked synaptic vesicles (gray spheres), along with AZ structures termed “pegs” (orange), “beams” (purple), and “ribs” (green). (**D**) The distribution of the AZ proteins bassoon (green) and P/Q-type VGCCs (magenta) at the mouse NMJ as revealed by STED super-resolution microscopy. (**E**) The distribution of the AZ proteins bassoon (green) and piccolo (magenta) at the mouse NMJ as revealed by STED super resolution microscopy. (**F**) A combined proposed overlay of all three AZ proteins (Bassoon, Piccolo, and P/Q-type VGCCs) onto the AZ fine structure based on STED super-resolution imaging. These data lead to the hypothesis that the “pegs” identified in electron microscopy tomography models from panel C represent P/Q-type VGCCs (orange), the “ribs” represent Bassoon (green), and the “beams” represent Piccolo (purple). C-F are adapted from [39] (**G**) The distribution of quantal content values determined from a population of mouse NMJs. Inset shows a sample miniature endplate current (mEPC; left) and a sample AP-triggered endplate current (EPC; right). (**H**) The distribution of AZ numbers counted from a population of mouse NMJs. Adapted from [5].

Active zones (AZs) are specialized structures on the presynaptic plasma membrane of synapses where neurotransmitter release occurs [3,32]. Early imaging studies showed that AZs contain docked synaptic vesicles and numerous intramembranous particles (Figure 2) [33,34,35,36]. These intramembranous proteins are hypothesized to include structural and functional proteins necessary for synaptic vesicle exocytosis; most prominently, the voltage-gated calcium channels. The mouse NMJ has a “pretzel” shape with small AZs (Figure 2). Each AZ contains, on average, a single row of two docked synaptic vesicles surrounded on both sides by double rows of intramembranous particles, containing approximately 20 particles in total (Figure 2) [10,36]. The average mouse NMJ contains approximately 700 of these AZs [5,37,38], each placed approximately 500 nm apart [37]. During a single AP, the mouse NMJ releases approximately 160 vesicles of neurotransmitters (Figure 2). This corresponds to a 0.22 probability of release from any given AZ during an AP, and, assuming two docked synaptic vesicles per AZ, a 0.11 probability of release per docked synaptic vesicle [5]. 

Super-resolution microscopy was used to study the distribution of active zone-specific proteins at mammalian NMJs. Stimulated emission depletion (STED) microscopy revealed the punctate staining of P/Q-type VGCC and Bassoon in nerve terminals, with the two proteins co-localizing together (Figure 2D) [39], consistent with their direct interaction [40]. Bassoon is a large scaffolding protein that contributes to the cytomatrix assembled at the AZ. The P/Q-type VGCC and Bassoon puncta aligned with bright linear areas of α-BTX staining representing junctional folds [41], which is consistent with the alignment of pre-synaptic AZs with junctional folds using electron microscopy and electron tomography [36,42]. The active zone protein Piccolo (another large scaffold-like cytomatrix protein at AZs) shares structural and functional similarities to Bassoon [43,44,45,46] and also overlaps with post-synaptic junctional folds [39]. STED microscopy demonstrated that Bassoon and Piccolo did not overlap in their distribution within adult mouse NMJs but instead were localized side-by-side in a Piccolo-Bassoon-Piccolo sandwich pattern (Figure 2E) [39]. This can represent a functional unit in the AZ structure characterized by electron microscope tomography [36]. Hypothetically, the AZ material macromolecules visualized in models based on electron tomography described as “pegs” are transmembrane proteins and may include P/Q-type VGCCs, while the “ribs” include Bassoon, and the “beams” represent Piccolo [39,47] (Figure 2F). Further speculation on the possible protein identities and contributions to the AZ macromolecules at the NMJ has been recently reviewed [48]. The additional mapping of super-resolution microscopy results onto AZ models developed based on EM tomography may yield important insights into AZ organization in both healthy and disease states. 

## 4. Structure-Function Relationships in the NMJ

It is clear from a comparison of AZ structures across many synaptic types that there are many ways to assemble the components required to couple presynaptic AP activity with chemical transmitter release [49]. One important question is as follows: How does the synaptic function depend on the particular manner by which AZs are organized or built? 

At the frog NMJ, Propst and Ko [50] combined electrophysiological recordings with the freeze fracture of identified NMJs and concluded that AZ size and spacing are better indicators of transmitter release than total NMJ size. Furthermore, Herrera et al. [51] used freeze fracture techniques to compare naturally occurring differences in synaptic strength between different types of muscles and found that NMJs with stronger transmitter release have significantly larger active zones. In addition, Herrera et al. [52] experimentally altered the magnitude of transmitter release in a long-lasting manner using contralateral denervation and concluded that changes in AZ size act as the structural basis for long-term changes in synaptic function. In addition to the total size of these AZ structures being important for synaptic function, there is also evidence that acutely disrupting the highly ordered structure can have a significant effects on transmitter release characteristics. After a three-hour exposure to a very low level of extracellular calcium (0.1 nM, which breaks divalent-dependent adhesion interactions in the synaptic cleft that are hypothesized to help hold AZ components in place), Meriney et al. [53] used freeze fracture to show that frog NMJ AZs broke into pieces and also displayed some dispersion of AZ proteins. When these disrupted AZs were returned to normal extracellular calcium to measure transmitter release, the AZ disruption was maintained for a long enough period to document a slight reduction in the magnitude of transmitter release, and a strong increase in short-term synaptic facilitation. These experiments demonstrated that disrupting AZ organization can profoundly affect synaptic function. Furthermore, these experiments isolate the effects of AZ-zone disruption on synaptic function, which is relevant in the consideration of the impact of various effects of the neuromuscular disease pathology of Lambert-Eaton Myasthenic syndrome (LEMS) on synaptic functions (see below). 

## 5. Computer Modeling of Active Zone Structure and Function

The impact of AZ organization has also been predicted using Monte Carlo Cell (MCell) to simulate diffusion and reaction events within the motor nerve terminal to model synaptic AZ anatomy and microphysiology. MCell is a stochastic particle-based diffusion-reaction simulator that can model biological systems with arbitrarily complex 3D geometries [54,55,56]. In MCell models of the NMJ AZs, an AP waveform is used to cause VGCCs to open according to a Markov-chain ion channel gating scheme. Calcium ions emanating from open VGCCs then diffuse into the nerve terminal space and can bind to the calcium buffer or calcium sensor proteins on synaptic vesicles.

Homan et al. [57] varied the density and distribution of VGCCs in a refined MCell model of the frog AZ (based on previously developed models of the frog AZ [58,59]) and demonstrated that these manipulations were predicted to significantly alter synaptic function (including the magnitude of transmitter release and the synaptic delay). By studying the impact of specific and systematic changes in AZ organization and VGCC density, Homan et al. [57] provided a foundation for further MCell modeling studies investigating the importance of AZ organization on synaptic function. Subsequently, Laghaei et al. [5] used MCell to compare the AZ organization in frog and mouse NMJs, each of which use similar AZ elements, but arrange them in different patterns. Frogs possess a very long (~1 μm) double row of proteins in their AZ array with 20–40 docked synaptic vesicles on the sides of these long arrays of proteins, while mice have very short arrays of AZ proteins (~150 nm) and two docked synaptic vesicles between the AZ protein arrays (see Figure 2). By simply rearranging the frog AZ elements into a mouse AZ organization (without changing any of the properties of the elements), Laghaei et al. [5] showed that their MCell models could recapitulate the known differences in short-term synaptic plasticity between these two synapses (frog NMJs facilitate strongly, while mouse NMJs have very little short-term synaptic plasticity). Furthermore, MCell models of the mouse AZs models are able to match many experimental electrophysiological results, including the probability of release per AZ of 0.22, a paired pulse facilitation that is relatively unchanged by changes in the inter-spike interval, and the log–log ratio between extracellular calcium and transmitter release (also known as the calcium-release ratio) [5]. These studies highlight the importance of AZ organization and structure in synapse functions.

## 6. Lambert-Eaton Myasthenic Syndrome

LEMS is an autoimmune-mediated neuromuscular disease in which the immune system attacks proteins in the NMJ AZ (especially the AZ voltage-gated calcium channels). LEMS is a rare disease with a prevalence of 3.4 cases per million people [8], and is often considered as a paraneoplastic syndrome because 50–60% of cases are associated with small-cell lung carcinoma [60]. In rare cases, LEMS has been associated with other malignancies such as non-small cell lung carcinoma [61] and prostate carcinoma [62]. Paraneoplastic instances of LEMS tend to be associated with older, male patients with a long-term history of smoking, whereas idiopathic LEMS patients tend to be younger and are more likely to be female. In almost all paraneoplastic LEMS cases, LEMS symptoms precede the diagnosis of small-cell lung carcinoma, and LEMS patients are routinely screened for lung cancer after their LEMS diagnosis [8]. The paraneoplastic relationship of LEMS with small-cell lung carcinoma is due to the fact that small-cell lung carcinomas are neuroendocrine in origin [63] and tend to overexpress VGCCs as well as other AZ proteins [64]. Thus, paraneoplastic LEMS is a result of the immune system response to the tumor [65].

The LEMS-mediated decrease in the number of voltage-gated calcium channels leads to a reduced calcium influx during an AP, and a subsequent reduction in the amount of neurotransmitters released [66]. This reduction in transmitter release results in muscle weakness which significantly limits the daily living-related activities of patients. Specific changes to the AZ structure and function in the LEMS disease state underlies the root pathophysiological cause of the disease symptoms. The presence of antibodies targeting AZ P/Q-type VGCCs is thought to support a LEMS diagnosis [7], but is not a complete explanation of the immune nature of LEMS. Although anti-P/Q-type VGCCs are the most common antibody reported, they are not present in 5–20% of LEMS patients [67,68,69,70]. This suggests that the immune nature of LEMS is more complicated than simply the presence or absence of P/Q-type VGCC antibodies. In fact, LEMS patients have been shown to produce auto-antibodies to a variety of presynaptic proteins, including synaptotagmin [68] and M1-type presynaptic muscarinic acetylcholine receptors [71].

## 7. The Passive Transfer Mouse Model for LEMS

Because LEMS is an antibody-mediated disease, and because many of the proteins targeted by LEMS are well-conserved between species, LEMS can be passively transferred to mice by repeatedly injecting them with serum from human LEMS patients [9,72]. The creation of these LEMS mice has facilitated a wide variety of investigations into the neuromuscular pathophysiology of LEMS. 

**Figure 3:**
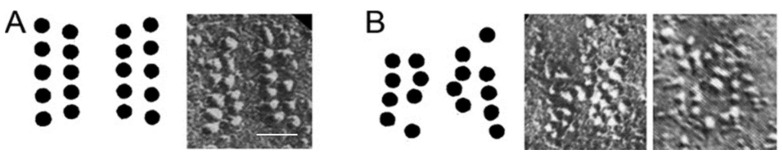
Freeze fracture of control and LEMS NMJs AZs showing AZ disruption after LEMS passive transfer to mice. (**A**) Representative control mouse AZ organization (left: diagram, right: freeze fracture replica, scale = 50 nm). (**B**) Representative LEMS-modified AZ organization (left: diagram of protein organization, right: two example AZs from nerve terminals of mice passively transferred with LEMS serum) Adapted from Nagel et al. [75]; Fukuoka et al. [10]; Fukunaga et al. [9].

Early freeze fracture electron microscopy studies of biopsied muscle tissue from human LEMS patients showed a decrease in the number of presynaptic AZs and a disorganization of particles in the remaining AZs (Figure 3) [73]. Similar results were found using freeze fracture electron microscopy in presynaptic terminals of LEMS-model mouse NMJs [9,10]. This disruption of the AZ structure was interpreted to be due to the antibody-mediated loss of P/Q-type VGCCs, which could explain the muscle weakness seen in LEMS patients. This hypothesis is further supported by evidence that the P/Q-type calcium current in LEMS mice is 30–40% less than in controls [66,74]. However, the lateral displacement of the remaining P/Q-type calcium channels may cause a further reduction in transmitter release, as the movement of VGCCs away from the calcium-sensing protein reduces their effectiveness. Therefore, disruptions in AZ organization may also play an important role in LEMS pathophysiology.

Blocking the P/Q-type VGCCs in a healthy mouse NMJ will normally completely block neurotransmitter release [76,77], suggesting that under healthy conditions, P/Q-type VGCCs are the only type of VGCC close enough to the vesicles in the AZ to induce transmitter release. However, in LEMS mice, other VGCC types (primarily L-type) were found to contribute to transmitter release [66,74,78]. This is generally thought to be a compensatory attempt by the motoneuron to increase transmitter release by overexpressing VGCC types. Despite this compensation, LEMS mouse NMJs release 60–75% less neurotransmitter than healthy controls [9,78,79,80,81]. Since L-type VCGGs do not contain the synaptic protein interaction (synprint) site that is necessary to associate with AZs, they are likely positioned outside of the AZ [82,83,84]. Indeed, preliminary experiments on LEMS passive-transfer mice found that the contribution of the L-type channel to the release of transmitters is blocked by the addition of low concentrations of the fast-acting calcium buffer BAPTA (1,2-bis(o-aminophenoxy)ethane-N,N,N′,N′-tetraacetic acid), suggesting that the L-type channels are not within close proximity to the calcium-sensing proteins in the AZs [85]. 

Interestingly, the injection of serum into mice from LEMS patients without detectable P/Q-type antibodies was successful in passively transferring LEMS symptoms to these mice, indicating that seronegative LEMS antibodies can still mediate the passive transfer of disease symptoms [86]. Furthermore, anti-VGCC antibody titers were not found to be predictive of long-term disease outcomes [87]. Therefore, LEMS auto-antibodies are likely to target antigens other than simply P/Q-type VGCCs, and the effects of these other antigens (i.e., synaptotagmin and muscarinic receptors; [68,71]) may contribute to the pathophysiology of LEMS.

## 8. Conclusions and Future Directions

**Figure 4:**
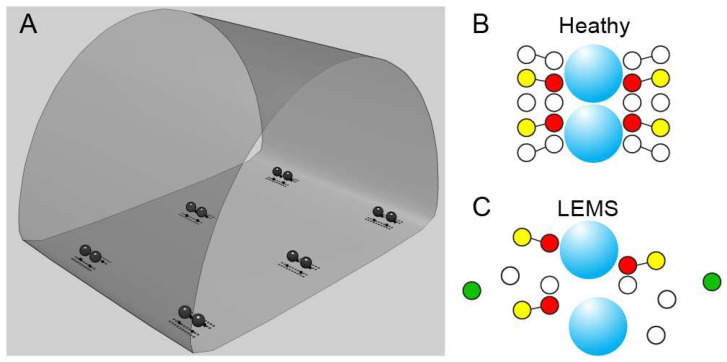
Diagrams of MCell models that can be constructed of mouse AZs in healthy and LEMS conditions. (**A**) Diagram of a mouse NMJ MCell model environment that contains 6 AZs. The 3-dimensional enclosure depicts a portion of the motor nerve terminal with black spheres representing docked synaptic vesicles within each AZ. Dots adjacent to the docked synaptic vesicles represent AZ proteins (gray dots) and P/Q-type VGCCs (black dots). (**B**) Diagram of a hypothesized healthy AZ based on predictions for the number and position of docked synaptic vesicles (blue spheres), P/Q VGCCs (red circles), calcium-activated potassium channels (yellow circles), and other unknown AZ proteins (white circles). (**C**) Diagram of a hypothesized LEMS AZ based on predictions for the number and position of docked synaptic vesicles (blue spheres), P/Q VGCCs (red circles), calcium-activated potassium channels (yellow circles), L-type VGCCs (green circles), and other unknown AZ proteins (white circles). Panel A is modified from Laghaei et al. [5].

There remains limited information on the structure and function of AZs within human NMJs. The few reports that have been published document that human NMJs are smaller than mouse NMJs, but possess many of the same features, proteins, and overall organization [88,89]. The development of mouse AZ models provides the background for exploring neurological disease states, and the pathophysiology of LEMS lends itself to exploration using MCell models. LEMS is often characterized as a disease of presynaptic VGCCs [66,74]. However, because it is known that other AZ changes occur in LEMS, including disruptions in the organization and alignment of AZ proteins [10], a compensatory upregulation of L-type VGCC subtypes outside of the AZs [74,78], and a reduction in the number of AZs [9], MCell modeling will be a useful tool to explore the impact of these changes (see Figure 4). If AZ changes beyond the removal of VGCCs from the AZ were to be implicated in LEMS pathophysiology, an in-depth characterization of autoantibody diversity in LEMS patient serum would be warranted, as well as super-resolution microscopy of the disruption of other proteins in LEMS-modified AZs. LEMS presents a unique opportunity to better understand the AZ structure and function in a pathological framework. A detailed understanding with regard to the range of autoantibodies generated across a range of patients and their impact on AZs is still required, as well as an investigation of the specific changes in AZs that lead to particular features of LEMS. In both healthy and diseased states, a more detailed understanding of the relationships between AZ composition, AZ organization, and synaptic functions would be beneficial.

Beyond pathological studies of neurological disease, basic questions remain. Many years of studying the mammalian NMJ AZ have led to an appreciation for some of the proteins that are expressed, and the functional consequences of the highly ordered structure of this transmitter release site. It is now clear that the strength and reliability of the neuromuscular synapses derive from the collective function of a very large number of individual release sites. However, there remain several very important unresolved issues. These include (1) how different individual AZs contribute to transmitter release under different conditions—both physiological and pathological, (2) a more detailed understanding of the specific proteins that underlie the ordered structure revealed by electron microscopy tomography studies, and (3) a better understanding of the potential heterogeneity in the protein make-up of individual AZs that might lead to heterogeneity in function.
